# Nuclear myosin VI cooperates with actin to promote transcriptional cluster formation at androgen receptors

**DOI:** 10.1016/j.jbc.2025.111088

**Published:** 2025-12-22

**Authors:** Irma M. Jayawardana, Justus M. Fleisch, Julian Knerr, Hong Wang, Manuel Holst, Stefan Tholen, Oliver Schilling, Robert Grosse

**Affiliations:** 1 Institute of Experimental and Clinical Pharmacology and Toxicology, Medical Faculty, University of Freiburg, Freiburg, Germany; 2 Spemann Graduate School of Biology and Medicine (SGBM), University of Freiburg, Freiburg, Germany; 3 Centre for Integrative Biological Signalling Studies - CIBSS, University of Freiburg, Freiburg, Germany; 4 Institute for Surgical Pathology, Medical Center - University of Freiburg, Medical Faculty - University of Freiburg, Freiburg, Germany; 5 Proteomics Platform - Core Facility (ProtCF), Medical Center - University of Freiburg, Medical Faculty - University of Freiburg, Freiburg, Germany

**Keywords:** Actin dynamics, androgen receptor, high resolution live-cell imaging, Lattice SIM, myosin VI, nuclear actin

## Abstract

Steroid hormone receptors are ligand-binding transcription factors essential for mammalian physiology. The androgen receptor (AR) binds testosterone mediating gene expression for sexual, somatic, and behavioral functions and is involved in various conditions, including androgen insensitivity syndrome and prostate cancer. Our previous work revealed the actin-dependent formation of transcriptional hubs consisting of the AR, the mammalian formin disheveled-associated activator of morphogenesis 2 (DAAM2) and active RNA Polymerase II (RNA Pol-II). Of note, highly dynamic nuclear F-actin polymerization by DAAM2, directly at the AR is essential for androgen signaling. To better understand actin-driven AR transcriptional activity, we turned our interest to the unconventional myosin VI, which was previously proposed to be involved in RNA Pol-II transcription. Indeed, dihydrotestosterone-dependent mass spectrometry of immunoprecipitated eGFP–myosin VI identified the AR as a prominent associator. Consistent with this, structured illumination microscopy in prostate cancer cells revealed signal-dependent nuclear enrichment of myosin VI, which localized in close proximity to AR as well as RNA Pol-II clusters and the actin nucleator DAAM2. Using live-cell structured illumination microscopy imaging, we directly visualized a ligand-dependent dynamic association between AR, myosin VI, and nuclear actin, revealing their spatially coordinated reorganization at AR clusters. Pharmacological inhibition of actin polymerization or inhibition of the myosin VI motor domain disrupted the formation of AR-related transcriptional clusters. Furthermore, reporter gene analysis and proliferation assays supported a critical role for myosin VI in AR signaling. Our findings thus uncover myosin VI as an essential regulator for the spatial organization of androgen-dependent transcription.

Nuclear actin dynamics are essential for maintaining nuclear architecture such as chromatin organization, preserving genome stability, and regulating transcription (1, 2, 3, 4). Hence, it was previously demonstrated that transient and signal-regulated nuclear F-actin structures respond rapidly to developmental and environmental cues, thereby coordinating multiple cellular processes. For example, following mitosis, actin polymerization facilitates nuclear expansion and chromatin decompaction (5, 6) and serves as a key link between extracellular G-coupled receptors signals to chromatin dynamics (7, 8). Additionally, nuclear actin dynamics maintain genome organization (9), mediate DNA repair mechanisms (10, 11), and support genome integrity during replication stress response (12, 13, 14) or to act as a scaffold for nuclear stiffness under conditions of mechanical stress and nuclear envelop rupture (15). Interestingly, nuclear actin dynamics orchestrate transcriptional regulation through diverse molecular pathways: modulating the SRF signaling pathway (5, 16), controlling Yes-associated protein (YAP)/TAZ activity (17), or regulating cytokine gene expression during T-cell activation (18). Furthermore, nuclear actin itself also associates with RNA Polymerase II (RNA Pol II) *via* hnRNPU (19, 20, 21) as well as RNA Polymerase I (22). Nonetheless, the precise role of dynamic nuclear actin assembly and turnover in gene transcription remains insufficiently well understood.

Notably, efficient transcription depends on the spatial organization of RNA Pol II into membraneless compartments forming dynamic multiprotein hubs also termed transcriptional condensates for their dynamic and coalescing behavior (23, 24). Super-resolution microscopy in embryonic stem cells revealed that active RNA Pol II clusters at transcription sites (25). Notably, polymerized nuclear actin has been shown to support both the formation and stabilization of RNA Pol II clusters during active transcription (8, 26). This actin-dependent organizational principle extends to hormone-dependent transcriptional regulation, where we recently showed that formin-dependent highly dynamic actin polymerization is critical for assembling transcriptional hubs containing the formin disheveled-associated activator of morphogenesis 2 (DAAM2), androgen receptor (AR), and active RNA Pol II (27).

The AR is a ligand-dependent transcription factor that is activated upon binding to its primary ligands, 5α-dihydrotestosterone (DHT) and testosterone. As a member of the steroid hormone receptor family, once activated, the AR undergoes conformational changes that facilitate importin-mediated nuclear translocation (28), thereby initiating a cascade of transcriptional events essential for male development as well as the bone muscle, prostate, or the hemopoietic system (29). Particularly, in prostate cancer (PCa), AR signaling is a central driver of tumor growth. While androgen deprivation therapy (ADT) initially suppresses AR activity, many tumors progress to castration-resistant prostate cancer through mechanisms such as AR gene amplification, point mutations, and expression of constitutively active splice variants lacking the ligand-binding domain (*e.g.*, AR-V7) (30, 31, 32). Importantly, the AR itself can cluster to form functional correlates of biomolecular condensates (33, 34, 35). We recently demonstrated that nuclear actin polymerization directly at the AR mediated by DAAM2 is essential for the formation of transcriptional clusters (27). DAAM2 belongs to the formin family of cytoskeletal regulators (36). We could recently show that patient mutations in DAAM2, that fail to polymerize nuclear actin at AR, are causative for partial androgen insensitivity syndrome (27). Therefore, DAAM2 was identified as a novel AR coregulator (1). Still, the precise molecular mechanisms as well as molecular factors involved in nuclear actin–dependent AR signaling remain to be investigated.

Myosin VI was recently reported to contribute to RNA Pol II–dependent transcription (37, 38). Unlike conventional myosins, myosin VI is an unconventional motor protein that moves toward the minus end of actin filaments (39, 40) and is reported to perform multiple nuclear functions, such as stabilization of replication forks during DNA replication stress (41), support of nucleolar function and ribosome biogenesis (42, 43), or facilitation of chromosome movement (44). During transcription, myosin VI forms dense protein clusters that stabilize RNA Pol II and promote transcription initiation (38, 42, 45). While these nuclear functions are increasingly characterized at the molecular level, clinical studies have independently identified myosin VI overexpression in aggressive cancers, such as breast cancer and PCa (46, 47, 48). Notably, in PCa, myosin VI overexpression enhances tumor survival and contributes to resistance against enzalutamide, a state-of-the-art ADT drug (49). This led us to investigate the potential participation of myosin VI for transcriptional cluster formation during androgen signaling.

Here, we provide evidence for a functional link between the unconventional myosin VI and nuclear actin-dependent transcriptional cluster formation during androgen signaling. Using structured illumination microscopy (SIM), we demonstrate that androgen stimulation induces the formation of distinct myosin VI-AR nuclear clusters. Additionally, we observe hormone-dependent myosin VI clustering with actin and the AR, as well as with DAAM2 and RNA Pol II, suggesting a coordinated transcriptional hub assembly. Furthermore, our data indicate that the motor domain of myosin VI is required for efficient AR-driven transcriptional activity in lymph node carcinoma of the prostate (LNCaP) PCa cells. Collectively, these findings identify nuclear myosin VI as an essential component for transcriptional cluster formation during hormone-dependent AR signaling.

## Results

### AR is a prominent DHT-induced interactor of myosin VI in PCa cells

**Figure 1:**
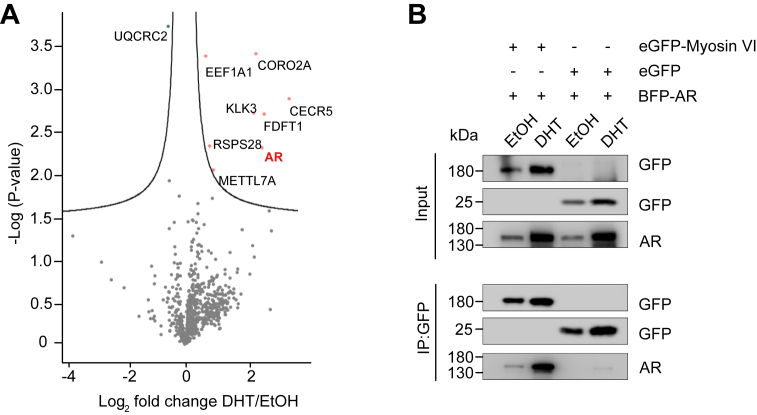
**Mass spectrometry data after immunoprecipitation reveal a hormone-dependent interaction between myosin VI and the AR.***A*, Volcano plot of proteins enriched in eGFP-myosin VI pull-down following DHT treatment in LNCaP cells. Volcano plot showing protein groups identified by mass spectrometry following eGFP-myosin VI pull-down in LNCaP cells treated with DHT versus EtOH. Difference between DHT and EtOH conditions is plotted against the −log_10_(*p*-value). Significantly enriched proteins under DHT treatment are shown in *pink*; decreased proteins are indicated in *blue* (*p* < 0.05). AR is indicated in *red*. *B*, Western blot analysis of GFP-Trap immunoprecipitates from HeLa cells co-expressing eGFP-myosin VI or eGFP alone with BFP-AR, following treatment with EtOH or DHT. Molecular weight markers are indicated (kDa). AR, androgen receptor; BFP, blue fluorescent protein; DHT, dihydrotestosterone; eGFP, enhanced GFP; SIM, structured illumination microscopy.

Given the emerging role of cytoskeletal components in nuclear signaling pathways, we sought to identify cytoskeletal regulators involved in AR-mediated transcription. Building on our previous findings (27), which demonstrated that nuclear actin plays a functional role in AR signaling, we turned our attention to nuclear myosin VI, a unique, reverse-direction motor protein known to interact with actin filaments and overexpressed in aggressive breast cancer and PCas (46, 48). To address this, we performed GFP-Trap immunoprecipitation in LNCaP cells expressing enhanced GFP (eGFP)-myosin VI or eGFP alone under vehicle (EtOH) or DHT treatment, followed by label-free mass spectrometry. Proteomic profiling revealed that DHT stimulation significantly altered the myosin VI interactome (Fig. 1*A*). Volcano plot analysis showed that multiple proteins were enriched in the DHT condition (*p* < 0.05, FDR > 0.1) (Fig. 1*A*). Among these, our myosin VI proteomics identified the core actin-regulator coronin 2A (CORO2A) (Fig. 1*A*) previously reported to be a component of the nuclear receptor corepressor complex, which is known to bind the AR (50, 51). Additionally, KLK3/prostate-specific antigen (PSA), a well-established downstream target of AR signaling (52), was prominently detected. Particularly, the AR itself emerged as one of the most robust and selective interactors under DHT treatment (Fig. 1*A*). To further examine this relationship, we performed co-immunoprecipitation experiments in HeLa cells overexpressing blue fluorescent protein (BFP)-AR and eGFP-myosin VI or eGFP alone. HeLa cells were chosen as a well-established, highly transfectable system, given that LNCaP cells grow very slowly and are notoriously difficult to transfect. Western blot analysis confirmed the presence of AR in the eGFP-myosin VI immunoprecipitates under both EtOH and DHT conditions, whereas no AR was pulled down in eGFP alone (Fig. 1*B*). Notably, AR coprecipitated more robustly with eGFP-myosin VI upon DHT stimulation, supporting a ligand-enhanced interaction (Fig. 1*B*).

### Myosin VI motor activity is required for DHT-induced nuclear clustering of AR and RNA Pol II

**Figure 2:**
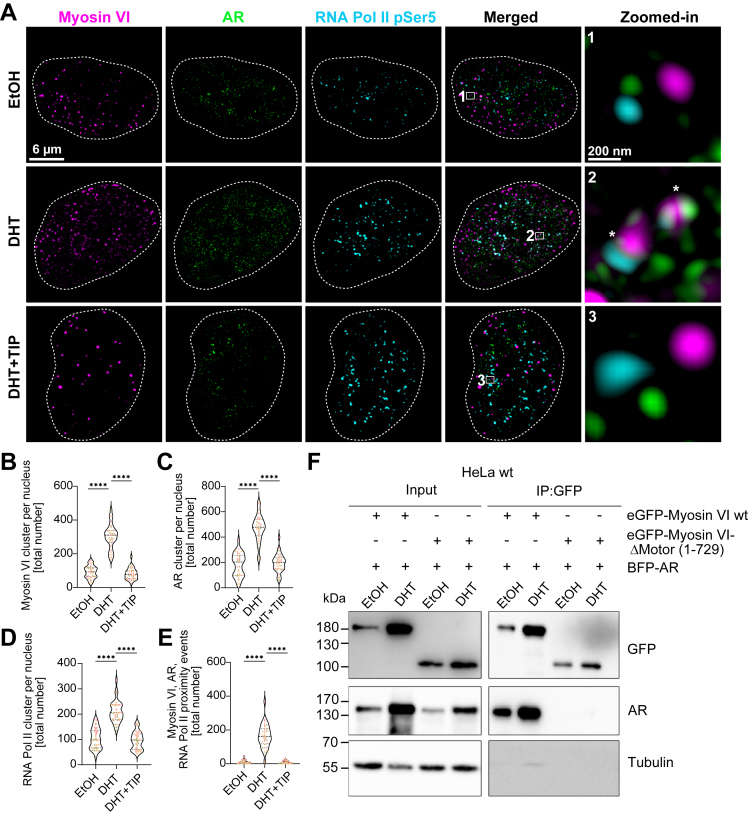
**DHT-dependent cluster formation of the AR, myosin VI, and RNA Pol II depends on the motor domain of myosin VI.***A*, super-resolution SIM imaging of LNCaP cells stained for AR (androgen receptor, *green*), myosin VI (*magenta*), and RNA Pol II pSer5 (*cyan*) under EtOH (*upper* panel), DHT (*middle* panel), and DHT + 2,4,6-triiodophenol (TIP) treatment (*lower* panel). For quantifications, nuclei were masked based on the DAPI signal and outlined with dashed lines; zoomed-in regions (*white* box) are shown separately. Proximity events are marked with asterisks. Scale bars represents 6 μm, 200 nm (Zoomed-in). Images are shown as maximum intensity projection (MIP). *B*–*D*, quantification of cluster number per nucleus for (*B*) myosin VI, (*C*) AR, (*D*) RNA pol II pSer5 are shown in three distinct colors, each representing an independent experimental replicate per each condition (EtOH, DHT, DHT + TIP). *E*, quantification of proximity events within 100 nm of AR, myosin VI, and RNA Pol II pSer5. *B*-*E*, violin plots show median and interquartile ranges from 10 cells per condition per biological replicate (n = 3). Triplicate samples shown in three distinct colors, each representing an independent experimental replicate. One-way ANOVA with Tukey’s multiple comparisons was used for statistical analysis; ∗∗∗∗*p* < 0.0001 (*B*–*E*). *F*, Western blot analysis of HeLa WT cells transfected with eGFP-myosin VI WT or eGFP-myosin VI-ΔMotor (1–729) and BFP-AR. Input and immunoprecipitation (IP) samples were probed for GFP, AR, and tubulin. Molecular weight markers are indicated (kDa). AR, androgen receptor; BFP, blue fluorescent protein; DHT, dihydrotestosterone; eGFP, enhanced GFP; SIM, structured illumination microscopy.

To investigate whether androgen signaling modulates the nuclear organization of myosin VI and the AR, we employed SIM, a super-resolution technique that enables high-speed, three-dimensional imaging with lateral and axial resolutions of ∼120 nm and ∼400 nm, respectively. Based on the well-established role of myosin VI in transcription (37, 38, 45), we evaluated whether androgen-driven transcription modulates its clustering alongside with AR and RNA Pol II CTD phosphorylation at serine 5 (RNA Pol II pSer5), which is a validated marker of transcriptional initiation (53). By specifically labeling RNA Pol II pSer5, we selectively visualized engaged RNA polymerases at sites of active transcription initiation rather than the total RNA polymerase pool. LNCaP cells were treated with EtOH, 24 h of DHT, or DHT in combination with 2,4,6-triiodophenol (TIP), a selective small molecule inhibitor targeting the motor domain of myosin VI (38, 54). Endogenous labeling was performed for myosin VI, AR, and RNA Pol II pSer5, with all three proteins exhibiting discrete nuclear clusters or spots clearly resolved by SIM (Fig. 2*A*). In the EtOH control, SIM resolved myosin VI, AR, and RNA Pol II pSer5 spots distributed sparsely throughout the nuclear volume outlined with dashed lines (scale bar = 6 μm) (Fig. 2*A*). Zoomed-in regions (scale bar = 200 nm) revealed well-separated individual clusters (Fig. 2*A*). Of note, DHT stimulation dramatically increased the number of myosin VI, AR, and RNA Pol II pSer5 clusters (Fig. 2*A*), which also exhibited clustering (Fig. 2*A*, white asterisks). However, following a 1 h TIP treatment after 24 h DHT, the clusters distributed similarly as in EtOH (Fig. 2*A*), with zoomed-in regions showing predominantly discrete spots and reduced clustering (Fig. 2*A*). More interestingly, TIP also dispersed the AR clusters (Fig. 2*A*), highlighting its pronounced effect on dynamic AR reorganization.

To confirm our observations, we quantified the total number of myosin VI, AR, and RNA Pol II pSer5 clusters per nucleus (Fig. 2, *B*–*D*). This showed that DHT stimulation markedly increased the total number of nuclear clusters for all three proteins—myosin VI, AR, and RNA Pol II—compared to EtOH treatment (Fig. 2, *B*–*D*). Nevertheless, this effect was entirely reversed upon 1 h TIP treatment (Fig. 2, *B*–*D*), suggesting that the clustering increase depends on intact myosin VI motor function. To further investigate the spatial coordination between these proteins, we performed proximity analysis at a 100-nm threshold. DHT significantly enhanced proximity events among myosin VI, AR, and RNA Pol II (Fig. 2*E*). Notably, this colocalization effect was reversed by TIP (Fig. 2*E*), reinforcing the role of myosin VI motor activity in assembling spatially coordinated nuclear transcriptional clusters.

To determine whether the spatial organization observed by super-resolution imaging reflects a direct role of myosin VI motor domain in the molecular association between myosin VI and AR, we extended our analysis with co-immunoprecipitation experiments. For this, we used a myosin VI truncation lacking the motor domain, eGFP–myosin VI-ΔMotor (1–729). In HeLa cells, we co-expressed BFP-tagged AR alongside either full-length eGFP–myosin VI or eGFP–myosin VI-ΔMotor (1–729) and performed immunoprecipitation to examine interaction under both EtOH and DHT conditions using GFP-Trap beads. Both full-length eGFP–myosin VI and eGFP–myosin VI-ΔMotor (1–729) were pulled down efficiently by GFP-Trap (Fig. 2*F*). Consistent with our imaging results, from fixed endogenous stainings, co-immunoprecipitated proteins confirmed a physical interaction between myosin VI and the AR (Fig. 2*F*). Importantly, this interaction was absent when the motor domain was deleted (Fig. 2*F*), indicating that the myosin VI motor domain is not only essential for spatial colocalization but also for molecular interaction with the AR.

### Myosin VI interacts dynamically with the AR and nuclear actin

**Figure 3:**
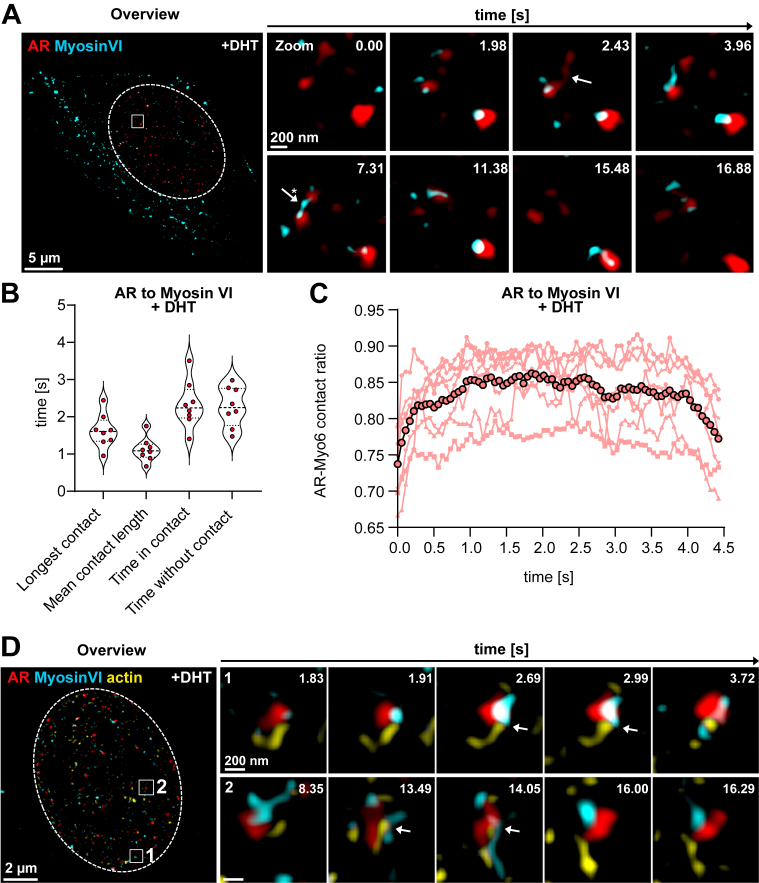
**Myosin VI interacts dynamically with the AR and nuclear actin.***A*, SIM-burst mode time-lapse imaging of a NIH3T3 cell cotransfected with AR-GFP (*red*) and Halo-myosin VI (*cyan*), acquired 16 h after DHT stimulation. The overview image shows AR and myosin VI localization within the nucleus (outlined by *white* box); the boxed region is magnified in the time-lapse series (0.00–16.88 s), highlighting coalescence between AR and nuclear myosin VI. Arrows indicate representative coalescence and bridging events. Scale bars represent 5 μm (overview), 200 nm (zoom). *B*, quantification of AR–myosin VI contact events in the presence of DHT. Violin plots show the distribution of longest contact duration, mean contact length, cumulative time in contact, and time without contact. Each *red* dot represents an individual AR trajectory; *violin* shapes indicate median and interquartile ranges. *C*, AR–myosin VI contact ratio over time following DHT stimulation. Individual traces (*pink* lines) represent single-cell measurements; the average trend (*black* line with *open* circles) shows a transient increase in contact ratio within the first 0.45 s. *D*, SIM-burst mode time-lapse imaging of a NIH3T3 cell cotransfected with AR-GFP (*red*), Halo-myosin VI (*cyan*), and nuclear actin chromobody-mCherry (*yellow*), acquired 16 h after DHT stimulation. The overview image shows AR, myosin VI, and nuclear actin localization within the nucleus (outlined by *white* box); regions 1 and 2 are magnified in the time-lapse series (1.83–16.29 s), showing myosin VI-AR cluster formation and nuclear actin moving towards the AR–myosin VI complex. Arrows indicate representative coalescence events. Scale bars represent 2 μm (overview), 200 nm (zoom). AR, androgen receptor; DHT, dihydrotestosterone; SIM, structured illumination microscopy.

Having identified a hormone-dependent interaction between myosin VI and AR, we next examined whether these proteins dynamically interact in living cells. To visualize this ligand-regulated association, we co-expressed AR-GFP and Halo-myosin VI in NIH3T3 mouse fibroblasts. Following androgen stimulation, we performed live-cell SIM imaging with enhanced temporal resolution by sliding processing (burst mode) (27). The processed videos resulted in a temporal resolution of around 18 frames per second and showed myosin VI–positive AR clusters upon androgen signaling (Fig. 3*A*, Movie S1). We observed the formation of myosin VI–AR clusters in the nucleus of the cells (Fig. 3*A*; Movie S1; nucleus outlined) that displayed coalescence events (Fig. 3*A*; Movie S1; zoom; white arrow) and fluctuated over time with transient contacts. Strikingly, myosin VI frequently localized to the interface between AR clusters and appeared to bridge them together (Fig. 3*A*; white arrow with asterisk; Movie S1). Moreover, we observed the AR–myosin VI clusters to move in a concerted fashion (Fig. 3*A*; Movie S1). To quantify these interactions, we implemented a MATLAB-based XTension for IMARIS specifically designed to analyze transient interactions in depth over time (Kiss and Run Analysis, Matthew Gastinger, Bitplane). We measured variables, such as the longest contact, mean contact length, time in contact, and time without contacts of the AR–myosin VI clusters over time (Fig. 3*B*). The longest individual contact events persisted up to 1.6 s, while the mean contact length across all clusters was approximately 1 s. On average, AR–myosin VI clusters remained in contact for approximately 50.6% of the imaging period, corresponding to a cumulative contact time of 2.33 s, and the intervals without contact averaging 2.27 s (Fig. 3*B*). Contact ratio analysis further demonstrated rapid fluctuations in the AR–myosin VI association showing that around 83% of the clusters were in contact over the imaging period (Fig. 3*C*).

To support and extend these findings, supporting information S2 presents additional quantitative outputs from the same analysis pipeline. Violin plots in panels S2A and S2B show the percent of surface contact and the number of contact events between AR and myosin VI clusters under DHT stimulation, respectively. Supporting information S2C shows representative trajectory overlays extracted from live-cell SIM imaging, where AR (red) and myosin VI (cyan) cluster paths are imaged over time. Specifically, AR trajectories are rendered in a cyan-to-magenta gradient, while myosin VI trajectories range from green to yellow. These color transitions reflect temporal progression and spatial displacement, where we visualized the dynamic colocalization and contact persistence.

To further observe the spatial dynamics of AR and myosin VI in the context of the nuclear architecture, we also sought to investigate how these interactions relate to nuclear actin dynamics. To this end, we co-expressed nuclear actin chromobody-mCherry (nAC-mCherry) together with AR-GFP and Halo-myosin VI in NIH3T3 fibroblasts and performed live-cell imaging following DHT stimulation. We observed that nuclear actin was exhibiting a dynamic behavior as previously described (27), and it was frequently moving toward AR–myosin VI clusters (Fig. 3*D*; Movies S2; S3). Of note, these movements often coincided with myosin VI sliding across the interface of AR clusters. Time-lapse sequences of selected nuclear regions (Fig. 3*D*; Boxes S1 and S2; Movies S2; S3) captured repeated coalescence and separation events, where nuclear actin filaments were approaching and contacting AR–myosin VI clusters. To complement the main imaging data, we included an additional overview in the supporting information (Fig. S1*A*) showing the same transfected cell (Fig. S1*A*, upper left box) alongside a neighboring nontransfected cell (Fig. S1*A*, lower right) under DHT stimulation. This comparative panel highlights the distinct nuclear organization induced by AR, nuclear actin, and myosin VI expression. Importantly, the transfected cell displayed AR–GFP, Halo–myosin VI, and nAC–mCherry within the nucleus (Fig. S1*A*; box; nucleus outlined by dashed lines), forming discrete clusters. In contrast, the AR-negative cell displayed cytoplasmic myosin VI localization further supporting nuclear-specific interactions upon androgen signaling (Fig. S1*A*, lower right). To further illustrate the subnuclear localization of each component, we included the individual fluorescence channels for AR, myosin VI, and actin (Fig. S1*A*, right panels) adjacent to the merged image (Fig. S1*A*, left panel).

### DHT treatment promotes interaction between myosin VI and DAAM2 in PCa cells

Given our prior findings that DAAM2 functions as an actin nucleator in LNCaP cells in response to androgens (27), we asked whether myosin VI may associate with DAAM2 to facilitate myosin VI–dependent assembly of the AR. To investigate this potential interaction, we employed endogenous proximity ligation assays (PLA) that enables the visualization of protein colocalizations within <40 nm using specific antibody pairs (17). To preserve nuclear architecture and retain actin cytoskeletal structures during fixation, cells were pre-extracted with CSK buffer prior to PLA processing. This step ensured the maintenance of intact F-actin networks.

**Figure 4:**
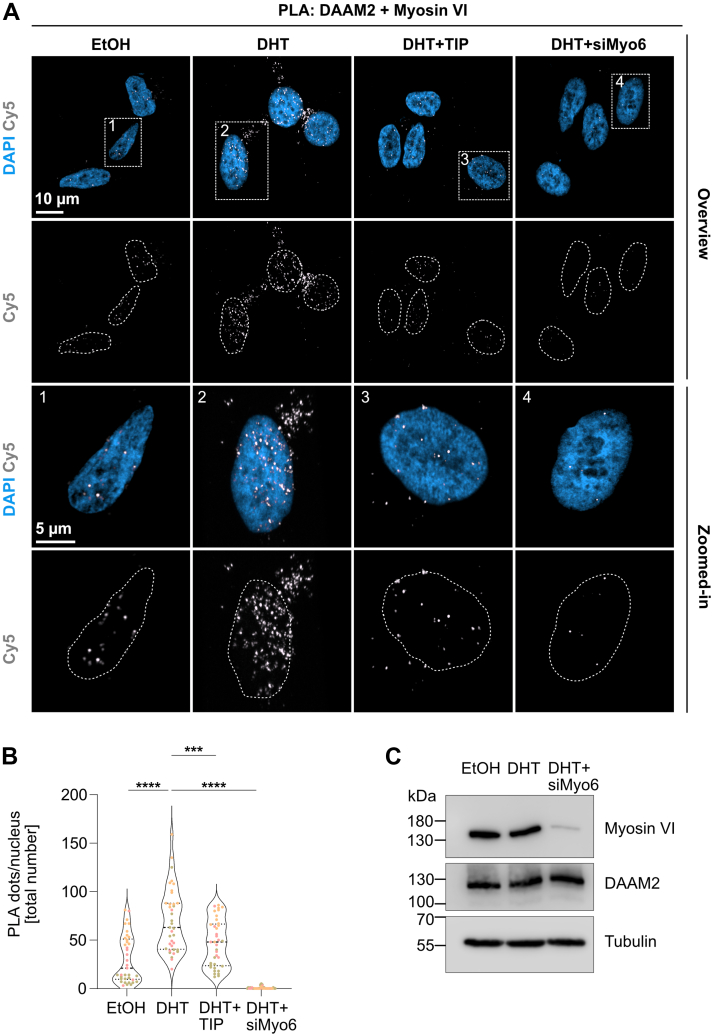
**Myosin VI interacts with the AR-cofactor DAAM2 upon AR signaling.***A*, immunofluorescence of proximity ligation assay (PLA) in LNCaP cells. DAPI (nuclei, *blue*) and Cy5 (PLA dots, *white*) are shown in cells treated with EtOH, DHT, DHT + TIP, and DHT + siMyo6 (negative control). Overview and zoomed-in images are indicated. Scale bars represent 10 μm (overview); 5 μm (zoomed-in). Images are shown as maximum intensity projection (MIP). *B*, quantification of nuclear PLA dots. Violin plots show median and interquartile ranges from 15, 10, 12 cells per condition (EtOH, DHT, DHT + TIP, DHT + siMyo6) per biological replicate (n = 3). Triplicate samples shown in three distinct colors, each representing an independent experimental replicate. One-way ANOVA with Tukey’s multiple comparisons was performed for statistical analysis; ∗∗∗∗*p* < 0.0001, ∗∗∗*p* < 0.001. *C*, Western blot of LNCaP cells treated with EtOH, DHT, or DHT + siMyo6, validating myosin VI knockdown and assessing DAAM2 expression. Tubulin serves as loading control. Molecular weight markers are indicated (kDa). AR, androgen receptor; DAAM2, disheveled-associated activator of morphogenesis 2; DHT, dihydrotestosterone; TIP, triiodophenol.

PLA analysis revealed distinct foci corresponding to myosin VI–DAAM2 complexes within the nucleus in LNCaP cells (Fig. 4, *A* and *B*) Notably, these PLA foci significantly increased upon stimulation with DHT (Fig. 4, *A* and *B*), suggesting enhanced nuclear proximity between the two proteins in response to androgen signaling. Interestingly, upon 1 h treatment with TIP, we observed a markedly significant decrease in the PLA signal (Fig. 4, *A* and *B*). Moreover, knockdown of myosin VI *via* siRNA resulted in a near-complete loss of PLA signal (Fig. 4, *A* and *B*), confirming the specificity and myosin VI dependency of the detected association. Western blot analysis confirmed efficient knockdown of myosin VI in siMyo6-treated cells. We also examined DAAM2 expression under EtOH, DHT, and siMyo6+DHT conditions, but no appreciable differences were detected (Fig. 4*C*). To validate the specificity of the PLA signal and ensure methodological rigor, we performed the complete panel of recommended control experiments according to the manufacturer’s kit instructions. These included antibody omission controls where each primary antibody was excluded separately or simultaneously. Additional negative controls were performed using myosin VI antibody paired with a noninteracting protein, YAP, while positive controls employed Disabled-2 (Dab2), a well-characterized myosin VI interactor. PLA assay showed no interaction between myosin VI and YAP, while significant amount of PLA foci was detected between myosin VI and Dab2 (Fig. S1, *B* and *C*), supporting the reliability and specificity of our PLA findings.

### Actin polymerization and myosin VI motor function cooperatively to control nuclear RNA Pol II clustering

**Figure 5:**
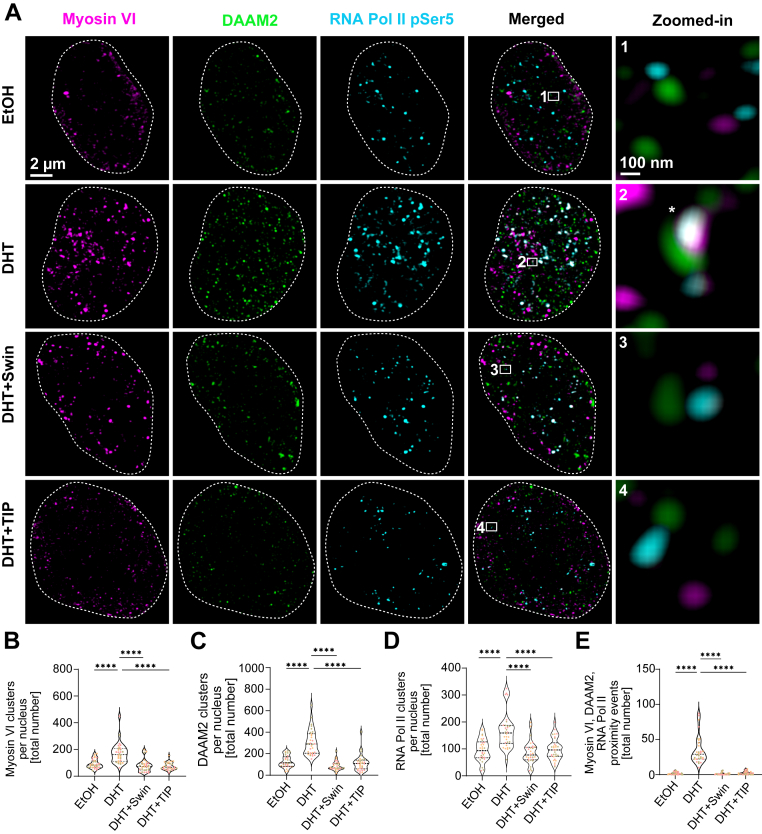
**DHT-dependent cluster formation of DAAM2, myosin VI, and RNA Pol II depends on the motor domain of Myosin VI.***A*, super-resolution SIM imaging of LNCaP cells stained for DAAM2 (*green*), myosin VI (*magenta*), and RNA Pol II pSer5 (*cyan*) under EtOH, DHT, DHT + Swinholide A (Swin), or DHT + TIP treatment. For quantifications, nuclei were masked, based on the DAPI signal and nuclei outlined with dashed lines; zoomed-in regions (*white* box) are shown separately. Proximity events are marked with *white* asterisks. Scale bars represent 2 μm (overview), 100 nm (zoomed-in). Images are shown as maximum intensity projection (MIP). *B*–*D*, quantification of cluster number per nucleus for (*B*) myosin VI, (*C*) DAAM2, (*D*) RNA pol II pSer5 are shown in three distinct colors, each representing an independent experimental replicate per each condition (EtOH, DHT, DHT + TIP). *E*, quantification of proximity events within 100 nm of DAAM2, myosin VI, and RNA Pol II pSer5. *B*–*E*, violin plots show median and interquartile ranges from 10 cells per condition (EtOH, DHT, DHT + TIP, DHT + Swin) per biological replicate (n = 3). Triplicate samples shown in three distinct colors, each representing an independent experimental replicate. One-way ANOVA with Tukey’s multiple comparison was used for statistical analysis; ∗∗∗∗*p* < 0.0001 (*B*–*E*). AR, androgen receptor; DAAM2, disheveled-associated activator of morphogenesis 2; DHT, dihydrotestosterone; SIM, structured illumination microscopy; TIP, triiodophenol.

To examine whether nuclear clustering of DAAM2, myosin VI, and RNA Pol II relies on F-actin polymerization, we employed SIM to achieve super-resolution imaging of LNCaP cell nuclei under the following conditions: EtOH, 24 h DHT stimulation, and DHT in combination with either TIP or Swinholide A (Fig. 5*A*). Swinholide A was used to sever F-actin and stabilize actin dimers (55). Cells were endogenously labeled for DAAM2, myosin VI, and RNA Pol II pSer5; nuclei were outlined by dashed lines. The immunofluorescence images revealed discrete nuclear puncta throughout the nuclei (Fig. 5*A*). Under EtOH control, DAAM2, myosin VI, and RNA Pol II pSer5 spots were evenly dispersed (Fig. 5*A*), and zoomed-in regions showed well-separated clusters (zoomed-in; Fig. 5*A*). Of note, DHT increased the number of DAAM2, myosin VI, and RNA Pol II pSer5 clusters (Fig. 5*A*, white asterisk) and induced their coalescence (Fig. 5*A*), indicating tight spatial coupling of DAAM2, myosin VI, and RNA Pol II pSer5. Remarkably, a 30-min Swinholide A treatment or 1-h TIP following 24 h DHT restored the DAAM2, myosin VI, and RNA Pol II pSer5 clusters to levels similar to EtOH and markedly reduced clustering (Fig. 5*A*). In addition, Swinholide A impaired the formation of myosin VI clusters indicative of an involvement of actin assembly (Fig. 5*B*). TIP treatment induced further perturbation of DAAM2 clustering (Fig. 5*A*). Next, we quantified the total number of clusters per nucleus of myosin VI, DAAM2, and RNA Pol II pSer5 under the indicated conditions and showed that DHT significantly elevated the total number of clusters per nucleus of DAAM2, myosin VI, and Pol II pSer5 compared to EtOH, and either Swinholide A or TIP fully reversed this increase (Fig. 5, *B*–*D*). Moreover, proximity analysis within a 100-nm threshold further showed a robust increase in DAAM2–myosin VI–RNA Pol II pSer5 proximity events upon DHT stimulation. However, when actin polymerization or myosin VI motor function was disrupted by Swinholide A or TIP treatments, the proximity events were remarkably reduced (Fig. 5*E*), indicating that both nuclear actin polymerization and the intact myosin VI motor activity are necessary for the clustering of myosin VI, DAAM2, and RNA Pol II clusters.

### Myosin VI motor activity is critical for androgen-dependent transcription and PCa cell proliferation

**Figure 6:**
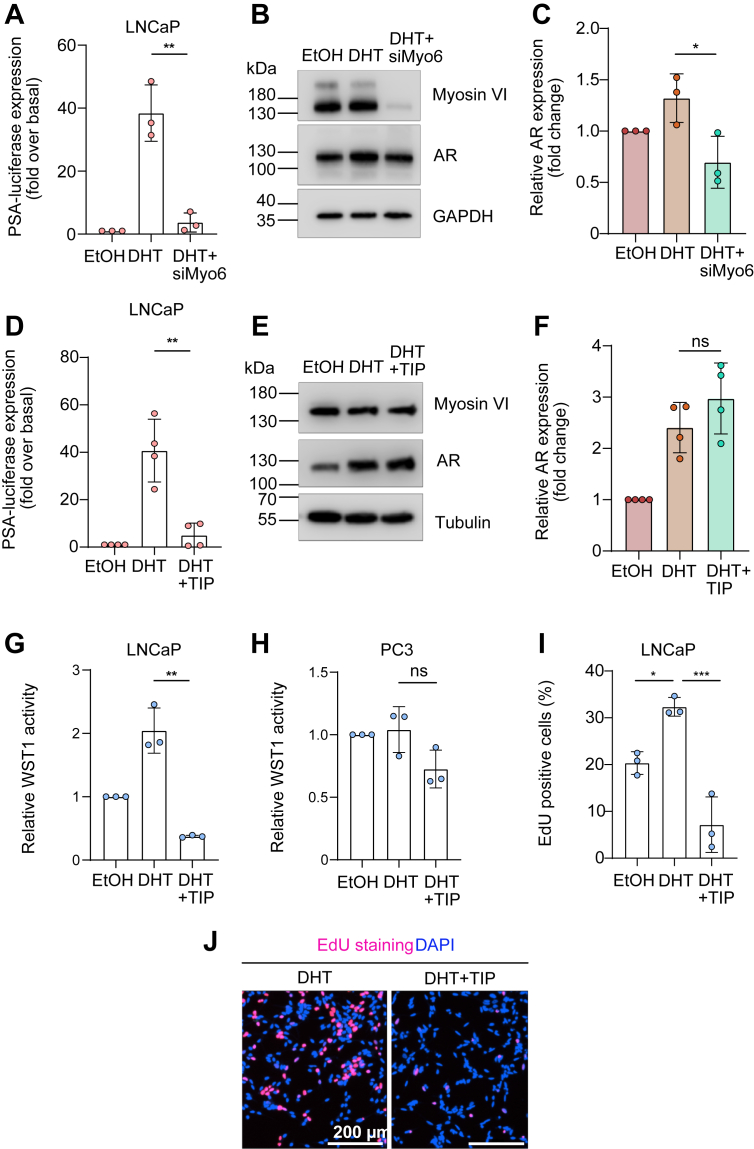
**The AR functions depend on DHT signaling and the motor domain of myosin VI.***A* and *D*, luciferase assay of PSA promoter activity in LNCaP cells treated with EtOH, DHT, and either siMyo6 (*A*, n = 3) or TIP (*B*, n = 4). All data are normalized to EtOH, shown as bar graphs with mean ± SEM. Statistical significance was determined using unpaired t-tests; ∗∗*p* < 0.01. *B* and *E*, Western blot analysis of myosin VI, AR, and loading controls (GAPDH or Tubulin) in LNCaP cells treated as in (*A*) and (*D*). Molecular weight markers are indicated (kDa). *C* and *F*, quantification of AR protein levels from Western blots in (*B*) and (*E*). DHT increases AR expression, which is reduced by siMyo6 (*C*) but not significantly affected by TIP (*F*). Data are normalized to EtOH and shown as mean ± SEM. Statistical significance was determined using unpaired t-tests; ∗*p* < 0.05, ns *p* > 0.05. *G* and *H*, WST-1 cell viability assay under EtOH, DHT, DHT + TIP treatment in LNCaP (*G*) and AR-negative PC3 cells (*H*). All data are normalized to EtOH, shown as bar graphs with mean ± SEM. Absorbance in WST-1 was measured at 450 nm, and significance in (*G*) and (*H*) was determined using unpaired t-tests; *∗∗p* < 0.01, ns *p* > 0,05. *I*, EdU proliferation assay in LNCaP cells under EtOH, DHT, DHT + TIP, analyzed by one-way ANOVA with Tukey’s multiple comparisons test; ∗*p* < 0.01, ∗∗∗*p* < 0.001. *J*, representative images of Click-iT Plus EdU assay in LNCaP cells under the indicated conditions and stained for DAPI (*blue*) and EdU (*magenta*). Scale bar represents 200 μm. AR, androgen receptor; DHT, dihydrotestosterone; TIP, triiodophenol.

To elucidate the role of myosin VI in AR-mediated transcription and PCa cell proliferation, we conducted a series of functional assays in LNCaP cells, which express endogenous AR. In reporter gene assays, PSA promoter–driven luciferase activity was used as a readout for AR transcriptional output. As expected, stimulation with DHT resulted in a ∼40-fold increase in luciferase activity compared to EtOH control (Fig. 6, *A* and *D*). Knockdown of myosin VI *via* siRNA significantly attenuated this induction, restoring luciferase activity to near-basal levels (Fig. 6*A*), indicating that myosin VI is required for full AR transcriptional activity. Similarly, pharmacological inhibition of myosin VI motor function using TIP led to a marked reduction in DHT-induced PSA promoter activity (Fig. 6*D*), underscoring the importance of myosin VI’s motor activity in facilitating AR-driven gene expression. To validate these findings, we also analyzed cell lysates by Western blot to assess protein expression under the same conditions. The expression of AR, myosin VI, and GAPDH were analyzed in siMyo6-treated cells (Fig. 6*B*), while AR, myosin VI, and tubulin were examined in TIP-treated cells (Fig. 6*E*). The quantification showed that siMyo6 treatment resulted in a modest statistically significant reduction in AR protein levels (Fig. 6*C*), whereas TIP treatment did not alter AR expression (Fig. 6*F*).

To assess the physiological relevance of myosin VI activity in androgen-mediated cell viability, we performed WST-1 assays in LNCaP and PC3 cells. In LNCaP cells, DHT significantly increased cell viability relative to EtOH, while TIP significantly decreased the enhanced cell viability from DHT (Fig. 6*G*). Interestingly, PC3 cells, which do not express AR, showed no significant changes in viability upon DHT or DHT + TIP treatments (Fig. 6*H*), confirming that the alteration of cell viability is AR-dependent.

Next, we evaluated the impact of myosin VI motor activity on cell proliferation using Click-iT EdU cell proliferation assays. DHT treatment significantly elevated the proportion of EdU-positive nuclei in LNCaP cells compared to EtOH (Fig. 6, *I* and *J*), indicating enhanced DNA synthesis and cell cycle progression. TIP cotreatment significantly reversed this effect, reducing EdU incorporation to baseline levels, as visualized in representative immunofluorescence images (Fig. 6, *I* and *J*). These results demonstrate that myosin VI motor activity is essential not only for AR transcriptional output but also for the downstream proliferative response to androgens.

## Discussion

This study elucidates the role of myosin VI in AR-driven transcriptional regulation and nuclear organization in PCa cells. We demonstrate that the AR is a hormone-induced interactor of myosin VI (Fig. 1, *A* and *B*), implicating this unconventional motor protein as a direct participant in androgen signaling. We found that the motor activity of myosin VI is required for the DHT-dependent nuclear clustering of AR and RNA Pol II (Fig. 2*A*) and AR activity in luciferase assay (Fig. 6, *A* and *D*), underlining its functional importance in hormone-dependent cluster formation and PSA gene expression.

We observed that myosin VI knockdown partially reduced AR protein expression under DHT stimulation, whereas TIP treatment did not (Fig. 6, *B* and *C*). Given that AR itself is a target gene of androgen signaling (56), (https://academic.oup.com/mend/article/7/7/924/2714837), this reduction may further underscore myosin VI requirement and involvement in AR transcriptional activity. The difference between knockdown and pharmacological inhibition of myosin VI could well be attributed to the different time scale of myosin VI inhibition (siMyo6 48 h/TIP 10 h).

These findings align well with prior research identifying myosin VI as a nuclear factor involved in RNA Pol II spatial organization and transcription (37, 38, 45). Notably, beyond its role in RNA Pol II clustering, we found that myosin VI motor activity contributes to the spatial organization of AR within the nucleus (Fig. 2, *A*–*D*), suggesting its fundamental role in the reorganization of transcriptional complexes as well as the formation of transcriptional clusters.

There are two possible mechanisms by which myosin VI regulates AR activity for transcription. Firstly, myosin VI contributes to the formation and spatial organization of dynamic membraneless compartments such as transcriptional hubs. By orchestrating the spatial organization of the AR and RNA Pol II within the nucleus (Fig. 2*A*), myosin VI could play a pivotal role in modulating or fine-tuning AR activity in response to hormone signaling. Secondly, myosin VI may influence cluster dynamics through local actin filament reorganization. Consistent with this idea, our live-cell imaging revealed that myosin VI frequently localized at the interfaces of AR clusters (Fig. 3*A*, Movie S1), coalescing and bridging them together, while nuclear actin filaments dynamically moved towards and across these complexes (Fig. 3, *A* and *D*; Movies S1–S3). The observed bridging and actin-driven movement suggest that myosin VI not only acts as a client recruited into AR clusters but also functions as a mobile scaffold that couples clusters to the nuclear actin network. Emerging evidence indicates that liquid-like droplets also regulate actin polymerization, balancing filament branching and bundling to influence cellular architecture and motility (57). For example, the actin-binding protein abLIM1 acts as a cross-linker enabling the self-assembly of highly connected actin networks through droplets formation (58). Our previous work showed that the formin and actin assembly factor, DAAM2, localizes to the nucleus, together with the AR, to form actin-dependent transcriptional droplets enriched in active RNA Pol II, structures essential for PSA gene expression (27). Consistent with this, disruption of nuclear F-actin structures or inhibition myosin VI motor activity diminishes clustering of myosin VI, DAAM2, and RNA Pol II (Fig. 5*A*), as well as the recruitment of AR (27) or DAAM2 (Fig. 5*A*) to RNA Pol II, indicating that both, nuclear F-actin as well as the motor domain of myosin VI are integral to the scaffolding architecture of AR transcriptional complexes.

While this manuscript was being revised, myosin VI was also implicated in regulating estrogen receptor activity, suggesting a broader role for myosin VI in steroid hormone receptor signaling (59).

The combined action of actin polymerization and the motor function of myosin VI in controlling nuclear clustering indicates a synergy mechanism in which mechanical forces and biochemical signals converge to modulate gene expression. However, the interplay between actin architecture, phase separation, and chromatin remains an open area of investigation.

Furthermore, our findings support myosin VI as a key mediator of androgen-dependent transcription and PCa cell proliferation. Notably, we showed that inhibition of the motor domain using TIP selectively impacted LNCaP cells, with no observable effect in PC3 cells, which lack the AR expression (Fig. 6, *G* and *H*). These different responses between LNCaP cells and PC3 cells show the dependance of the Myosin VI motor function on AR signaling and further validates its crucial role in hormone-driven transcriptional programs.

Myosins are an emerging target for drug development. Mavacamten is a recently approved drug by the US Food and Drug Admnistration (FDA) for the treatment of the hypertrophic cardiomyopathy and it is a first-in class targeted cardiac-specific myosin inhibitor (60). Given the important role of myosin VI motor activity in AR signaling and AR’s central role in PCa progression (61), targeting myosin VI—either by inhibiting its motor function or disrupting its interactions with AR and DAAM2—may offer a novel therapeutic strategy to attenuate AR target gene expression thereby suppressing tumor growth.

Moreover, our results, emphasizing the importance of myosin motor activity in transcription, raise broader questions about the role of motor proteins in nuclear organization and transcriptional control. While myosin VI has been extensively studied in the nucleus, other myosins and cytoskeletal regulators, that have been lately reported to be in the nucleus (62), may perform analogous functions. For instance, in breast cancer cells, both nuclear myosin I and myosin VI contribute to estrogen receptor α-mediated transcription and clustering (37, 59, 63). However, whether these functions are related to the motor domain activity still need to be elucidated.

In conclusion, our study identifies myosin VI as a central player in androgen signaling linking mechanical motor activity to nuclear architecture and transcriptional regulation. These findings deepen our molecular understanding on the regulation of AR activity by myosin VI and open new paths for therapeutic intervention in PCa. Nonetheless, they highlight the emerging significance of the actin nucleoskeleton in orchestrating gene expression, particularly in hormone-sensitive malignancies.

## Experimental procedures

### Cell culture

LNCaP cells (ATCC) were cultured in RPMI 1640 medium (Gibco) supplemented with 10% fetal bovine serum (FBS; Biochrome) and 100 U/ml penicillin–streptomycin (P/S) at 37 °C in a humidified atmosphere containing 5% CO_2_. PC3 cells (ACC 465, DSMZ) were maintained in a 1:1 mixture of RPMI 1640 (Gibco) and Ham’s F12 medium, supplemented with 10% FBS (Biochrome) and 100 U/ml P/S, under the same incubation conditions. HeLa or NIH3T3 (ATCC) cells were cultured in Dulbecco’s Modified Eagle medium (DMEM) High Glucose medium (anprotec) containing stable glutamine and sodium pyruvate, supplemented with 10% FBS (Biochrome) and 100 U/ml P/S, at 37 °C in 5% CO_2_. All cells were regularly tested for *mycoplasma* contamination using the VenorGem Classic *Mycoplasma* detection kit (Minerva Biolabs).

### Androgen stimulation and inhibitor treatments

For androgen stimulation experiments, cells were hormone-deprived for 24 h in their respective base media supplemented with 10% charcoal-stripped FBS (csFBS; Gibco, charcoal stripped, USDA-approved regions) and 100 U/ml P/S. Charcoal-stripped medium was prepared individually for each cell type: RPMI 1640 for LNCaP cells, a 1:1 mixture of RPMI 1640 and Ham’s F12 for PC3 cells, and DMEM High Glucose for HeLa and NIH3T3 cells. Following deprivation, LNCaP cells were treated with 10 nM nonaromatizable DHT (Sigma-Aldrich) or ethanol (EtOH) vehicle control for 24 h. Where indicated, cells were pretreated 24 h after DHT stimulation with either 25 μM TIP (no. 137723, Sigma-Aldrich) for 1 h or 100 nM swinholide A (actin polymerization inhibitor; no. 19611, Cayman Chemicals) for 30 min prior to fixation or lysis. For luciferase reporter and proliferation assays, TIP or Swinholide A was added 5 h post androgen stimulation and incubated overnight.

### Cloning and plasmid constructs

The full-length eGFP–myosin VI expression vector was generated by Gibson Assembly into the pCDNA3.1 backbone using the pCDNA3.1-Halo-MVI plasmid (gift from Christopher Toseland, University of Sheffield) as the myosin VI template. The eGFP fragment was amplified from pEGFP-N1 employing primers EGFPN1.FOR (5′-AGCTGGCTAGCATGGAATTCATGG TGAGCAAGGGCGAG-3′) and EGFPN1.REV (5′-GGATCGCCTGTTGTTGGCTCCTTGTAC AGCTCGTCCATGCC-3′). The myosin VI coding region was PCR-amplified using BB_MVI.FOR (5′-TGGACGAGCTGTACAAGGAGCCAACAACAGGCGATCC-3′) and BB_MVI.REV (5′-TCCTCGCCCTTGCTCACCATGAATTCCATGCTAGCCAG-3′). Fragments and linearized pCDNA3.1 were assembled with Gibson Assembly Master Mix (NEB), and resulting colonies were verified by Sanger sequencing.

A motor-domain deletion construct, pEGFP-myosin VI-ΔMotor (1–729), was kindly provided by Hans-Peter Wollscheid (Institute of Molecular Biology, Mainz). This plasmid lacks the N-terminal motor region of myosin VI and was employed in co-immunoprecipitation experiments.

pEGFP-N1 was used as control vector for co-immunoprecipitation experiments.

To generate the pCDNA-tagBFP-AR construct, the BFP coding sequence was amplified from the pEF-myc-NLS-BFP plasmid and substituted for the GFP sequence in the pCDNA-AR-GFP backbone using Gibson Assembly. The BFP insert was PCR-amplified using primers mTagBFP2.fwd (5′ttaaacttaagcttgccaccATGGAGCTGATTAAGGAGAACATGCACATGAAGCTG-3′) and TagBFP2.rev (5′-atggatccgagctcggtaccAAGCTTGTGCCCCAGTTTGCTAGGG-3′), which introduced overlapping sequences compatible with the backbone. The pCDNA-AR-GFP backbone was amplified using primers BB-AR.fwd (5′-GCA ACTGGGGCACAAGCTTggtaccgagctcggatccatggaagtgcag-3′) and BB-AR.rev (5′-ATGTTCTCCTTAATCAGCTCCATggtggcaagctta agtttaaacgctagccagc-3′), designed to exclude the GFP sequence and provide homology arms for insertion of the BFP fragment. Fragments and linearized pCDNA3.1 were assembled with NEB Gibson Assembly Master Mix, and resulting colonies were verified by Sanger sequencing.

### RNA interference

LNCaP cells (150.000 cells) were seeded in a 6-well plate and transfected the following day with either 30 pmol siMyo6 (Qiagen FlexiTube Hs_MYO6_5 (AGAGATAAGTTTATACGGGAA)) for myosin VI knockdown or siCTRL (Qiagen AllStars Negative Control (AATTCTCCGAACGTGTCACGT)) in Opti-MEM mixed with Lipofectamine RNAiMAX (Thermo Fisher Scientific). Cells remained in hormone-depleted conditions for a total of 24 h before androgen stimulation and were harvested 48 to 72 h after the initial transfection for downstream assays. Knockdown efficiency was confirmed by Western blotting.

### Immunoblotting

After 24 h DHT stimulation, LNCaP cells were lysed directly in 1 × Laemmli sample buffer and heated at 95 °C for 10 min to denature proteins. Equal volumes of each lysate were loaded onto 10% or 8% SDS-polyacrylamide gels for electrophoretic separation. Following SDS-PAGE, proteins were transferred to polyvinylidene fluoride membranes using a semidry transfer system (Power Blotter XL, Invitrogen). Membranes were blocked in 5% nonfat dry milk prepared in Tris-buffered saline with 0.1% Tween 20 detergent for 1 h at room temperature to prevent nonspecific antibody binding. Primary antibodies specific to the target proteins were incubated with the membranes overnight at 4 °C. The next day, membranes were washed and incubated for 1 h at room temperature with horseradish peroxidase (HRP)-conjugated secondary antibodies (1:3000): anti-Rabbit IgG, HRP-linked (Cell Signaling Technology, #7074) and anti-Mouse IgG, HRP-linked (Rockland, #310-703-002). Protein bands were detected using Amersham Imager 600 (GE Healthcare Life Sciences) and visualized using a high-sensitivity enhanced chemiluminescence substrate (SuperSignal West Femto Maximum Sensitivity Substrate, Thermo Fisher Scientific). The following primary antibodies were used: mouse monoclonal anti-myosin VI (1:1000; Sigma-Aldrich, M0691), mouse monoclonal anti-GAPDH (1:20.000; Millipore, 3587127), rabbit monoclonal anti-DAAM2 (1:1000; abcam, ab169527), rabbit monoclonal anti-alpha-tubulin (1:1000; cell signaling, 2125S), and mouse monoclonal anti-Androgen Receptor (1:1000; clone MU2560721; Biogenex).

### Quantification of Western blot data

Western blot band intensities were quantified using ImageJ (NIH). For each band, the area under the peak was calculated from the densitometric profile generated by the software. To correct for loading variability, target protein signals were normalized to the corresponding loading control (GAPDH or α-Tubulin). Normalized values were then expressed relative to the EtOH-treated control, which was set equal to 1. Quantifications were performed across at least three independent biological replicates, and data are presented as mean ± SD.

### GFP immunoprecipitation

LNCaP cells were transfected with either eGFP–myosin VI (MVI) or eGFP control plasmids using Lipofectamine 3000 (Thermo Fisher Scientific) according to the manufacturer’s protocol. Transfection was performed 24 h prior to treatment with EtOH or DHT. Cells were lysed in 1 × RIPA buffer prepared by diluting a 10 × stock solution containing: 200 mM Tris–HCl (pH 7.5), 1.5 M NaCl, 10 mM Na_2_EDTA, 10 mM EGTA, 10% NP-40, 25 mM sodium pyrophosphate, 10 mM sodium orthovanadate (Na_3_VO_4_), and 10 mM β-glycerophosphate. Protease inhibitor cocktail (Roche) was added to both the lysis buffer and washing buffer. Clarified lysates were incubated with GFP-Trap agarose beads (ChromoTek) for 1 h at 4 °C. Beads were washed four times with GFP-Trap washing buffer (10 mM Tris–HCl pH 7.5, 150 mM NaCl, 0.1% NP-40), supplemented with protease inhibitor cocktail. Bound proteins were eluted with 2 × Laemmli buffer and analyzed by SDS-PAGE followed by Western blotting.

HeLa cells were seeded in 10 cm dishes, 24 h later, transfected with pCDNA3.1-eGFP–myosin VI or pEGFP-myosin VI (ΔMotor 1–729) using Fugene HD (Promega) according to the manufacturer’s protocol. After an additional 24 h, cells were lysed as described above, GFP-Trap IP performed, and eluates analyzed by SDS-PAGE and Western blot. The following primary antibodies were used: mouse monoclonal anti-myosin VI (1:1000; Sigma-Aldrich, M0691), rabbit monoclonal anti-GFP (1:1000; clone D5.1; Cell Signaling Technology), rabbit monoclonal anti–α-Tubulin (1:1000; clone 15; Cell Signaling Technology), and mouse monoclonal anti-Androgen Receptor (1:1000; clone MU2560721; Biogenex).

### Mass spectrometry

For elution, proteins bound to beads were resuspended in lysis buffer (5% SDS, 50 mM triethyl ammonium bicarbonate (TEAB), pH 7.5) and incubated at 95 °C for 10 min. Samples were centrifuged at 13000*g* for 8 min and the supernatant used in the following steps. Proteins were reduced using 5 mM tris (2-carboxyethyl) phosphine hydrochloride (Sigma; 75259) for 10 min at 95 °C and alkylated using 10 mM 2-iodoacetamide (Sigma; I1149) for 20 min at room temperature in the dark. Following steps were performed using S-Trap micro filters (Protifi) following the manufacturer’s procedure. Briefly, first, a final concentration of 1.2% phosphoric acid and then six volumes of binding buffer (90% methanol; 100 mM TEAB; pH 7.1) were added. After gentle mixing, the protein solution was loaded to an S-Trap filter and spun at 2000 rpm for 0.5 to 1 min. The filter was washed three times using 150 μl of binding buffer. Sequencing-grade trypsin (Promega, 1:25 enzyme:protein ratio) diluted in 20 μl digestion buffer (50 mM TEAB) were added into the filter and digested at 47 °C for 1 h. To elute peptides, three step-wise buffers were applied: a) 40 μl 50 mM TEAB, b) 40 μl 0.2% formic acid in H2O, and c) 50% acetonitrile and 0.2% formic acid in H2O. The peptide solution was combined and dried in a SpeedVac.

Peptides were analyzed with the Evosep One system (Evosep Biosystems) coupled to a timsTOF fleX mass spectrometer (Bruker). Five hundred nanograms of peptides were loaded onto Evotips C18 trap columns (Evosep Biosystems) according to the manufacturer’s protocol. Peptides were separated on an EV1137 performance column (15 cm × 150 μm, 1.5 μm, Evosep) using the standard implemented 30 SPD method with a gradient length of 44 min (buffer A: 0.1% v/v formic acid, dissolved in H2O; buffer B: 0.1% v/v formic acid, dissolved in acetonitrile). The timsTOF fleX mass spectrometer was operated in the DDA-PASEF mode. MS and MS/MS spectra were acquired in an m/z range from 100 to 1700. Ion mobility resolution was set to 0.60 to 1.60 V s/cm over a ramp time of 100 ms and an accumulation time of 100 ms. The data-dependent acquisition was performed using 10 PASEF MS/MS scans per cycle with a near 100% duty cycle. An active exclusion time of 0.4 min was applied to precursors that reached 20,000 intensity units. The collision energy was programmed as a function of ion mobility, following a straight line from 20 eV for 1/K0 of 0.6 to 59 eV for 1/K0 of 1.6. The TIMS elution voltage was linearly calibrated to obtain 1/K0 ratios using three ions from the ESI-L TuningMix (Agilent) (m/z 622, 922, 1222).

Raw data were analyzed with MaxQuant (v 2.1.4.0) with the built-in Andromeda peptide search engine (64) allowing two missed cleavage sites, no variable modifications, and carbamidomethylation of cysteines as fixed modification. The match between runs option was selected. The Human-EBI-reference database was downloaded from https://www.ebi.ac.uk/ on July 22nd 2023. The sequence of GFP (GFP; Uniprot ID P42212) was added to the database. For label-free quantification, the MaxLFQ algorithm was applied using the standard settings. Only unique peptides were used for quantification.

### Perseus analysis

Quantitative proteomic analysis was conducted following the workflow as reported previously (65) with modifications to accommodate our experimental design. Protein Groups files generated from GFP-Trap pulldown experiments of LNCaP cells expressing eGFP-myosin VI or eGFP alone, with EtOH or DHT-treatment, were processed using Perseus (v2.1.3.0) (66). To ensure high-confidence data, potential contaminants and reverse hits were removed. Label-free quantification (LFQ) intensity values were log_2_-transformed to approximate a Gaussian distribution across the dataset.

Experimental comparisons were defined based on specific DHT-treated GFP-Trap pulldown *versus* control (EtOH-treated) conditions. Proteins were retained for downstream analysis if detected in at least three replicates within one of the two groups. Missing LFQ values were imputed using the “entire matrix” mode, with a width of 0.3 and a shift of 2.5. Differential enrichment analysis was performed using a two-sample *t* test comparing triplicates from corresponding DHT-treated and EtOH-treated conditions. In the GFP-Trap pulldown analyses, we applied a false discovery rate of 0.1 and an S_0_ value of 0.1. Volcano plots were generated using these statistical parameters to visualize significantly enriched proteins.

### Immunofluorescence

LNCaP cells were cultured on sterile 10 μg/ml fibronectin-coated glass coverslips in 24-well plates and grown to approximately 60 to 70% confluence. Cells were incubated in RPMI 1640 medium supplemented with csFBS for 24 h to deplete endogenous steroids. Subsequently, cells were treated with 10 nM DHT for an additional 24 h.

Prior to fixation, cells were exposed to 25 μM TIP for 1 h or to 30 min of Swin treatment under respective experimental conditions. After treatment, cells were fixed using 4% paraformaldehyde in PBS for 10 min at room temperature and permeabilized with 0.3% Triton X-100 in PBS for 5 min.

To block nonspecific antibody binding, cells were incubated with 5% bovine serum albumin (BSA) in PBS for 1 h at room temperature. Primary antibodies were diluted in 5% BSA blocking buffer and incubated overnight at 4 °C. After three PBS washes, fluorophore-conjugated secondary antibodies were applied for 1 h in a dark humidity chamber at room temperature. Nuclei were counterstained with DAPI (1 μg/ml; Sigma-Aldrich, D9542) for 5 min. Coverslips were mounted using ProLong Diamond Antifade Mountant (Invitrogen). The following primary antibodies were used: rabbit monoclonal anti-DAAM2 (1:200; Abcam, EPR10797(B)), mouse monoclonal anti-myosin VI (1:200; Sigma-Aldrich, M0691), and rat monoclonal anti-RNA Pol-II CTD phospho Ser5 (1:300; Active Motif, 61986), and rabbit monoclonal anti-Androgen Receptor (D6F11) (1:100; Cell Signaling Technologies, 5153). The following secondary antibodies were employed: Alexa Fluor 647-conjugated goat anti-rat IgG (H + L) (Invitrogen, Cat# A21235, Lot# 1890864), Alexa Fluor 568-conjugated donkey anti-mouse IgG (H + L) (Invitrogen, Cat# A10037, Lot# 1752099), and Alexa Fluor 488-conjugated chicken anti-rabbit IgG (H + L) (Invitrogen, Cat# A21441, Lot# 1796684). These antibodies were selected based on species compatibility and fluorophore requirements for multiplex imaging.

Imaging was performed using an ELYRA 7 structured-illumination microscope with 3D Lattice SIM capability (Zeiss), equipped with a × 63/1.4 NA oil DIC objective and a Pecon incubation chamber maintained at 30 °C for all samples. Image acquisition and processing were conducted using Zen Black software (Zeiss). SIM reconstruction was carried out utilizing the theoretical optical transfer function provided by the manufacturer, with Wiener filter strength set according to the “standard” end criterion. Imaging conditions and general analysis protocols followed those described in (27).

For 3D reconstruction and quantification of nuclear clusters, treated and stained cells were imaged as z-stacks with a 0.091 μm interval. Image stacks were processed with IMARIS v10.2.0 (Oxford Instruments) and displayed as maximum-intensity projections. Clusters analysis was performed in Imaris, enabling quantification of cluster number. Clusters smaller than 0.005 μm^3^ were excluded from cluster quantification due to axial resolution limitations. Brightness and contrast adjustments were applied uniformly across all images prior to quantification.

To assess 3D colocalization, clusters were rendered and analyzed based on minimum distance thresholds ranging from 0 to 100 nm, enabling high-resolution evaluation of spatial proximity. This method calculates the minimum Euclidean distance between the surfaces of objects in different fluorescence channels, providing high-resolution spatial proximity measurements without relying on intensity overlap. A distance threshold ranging from 0 to 100 nm was applied to define and quantify interactions between clusters rendered in 3D.

### Structured illumination live cell imaging

For live-cell imaging, NIH3T3 cells were seeded in DMEM (Gibco) supplemented with 10% FCS and P/S in 35 mm glass-bottom dish (Greiner) at 37 °C and 5% CO_2_. The following day, cells were cotransfected with nuclear actin chromobody-mCherry (nAC-mCherry), AR-GFP, and Halo-myosin VI in Opti-MEM (Thermo Fisher Scientific) using Lipofectamine 2000 (Thermo Fisher Scientific), following the manufacturer’s instructions. Five hours after transfection, the cells were hormone-deprived for 24 h in their respective base media supplemented with 10% csFBS (Gibco, charcoal stripped, USDA-approved regions) and 100 U/ml P/S. After 24 h, medium was changed to 10 nM DHT in DMEM supplemented with 10% charcoal-stripped FCS and P/S; 16 h after the addition of DHT, imaging was performed. Prior to imaging, Janelia Fluor JFX650 HaloTag Ligand (Promega) was diluted in 10 nM DHT containing DMEM supplemented with 10% charcoal-stripped FCS and P/S according to the manufacturer’s protocol.

### Quantification of surface association using the Imaris "Kiss and Run Analysis" XTension

#### Surface generation and preparation

Three-dimensional surface objects representing the cellular structures of interest were created within IMARIS v10.2.0 software (Oxford Instruments) using identical reconstruction workflow to ensure comparable surface detection between the two analyzed populations. Surface reconstruction was performed by setting intensity thresholds that determined which voxels from the original fluorescence microscopy images were included in the final surface objects.

### Ratio of AR surfaces contacting myosin VI surfaces

After surface generation within IMARIS v10.2.0 software as described above, the surfaces representing the AR were filtered for “Shortest distance to surface 2” below or equal of 0 nm, where surface 2 represents myosin VI. The number of AR surfaces in a shortest distance below or equal of 0 nm to myosin VI surfaces of each frame was divided by all AR surfaces of that frame.

### Contact event detection algorithm

Spatial association between the two surface populations was quantified further using the custom MATLAB-based "Kiss and Run Analysis" XTension (version 2, developed by Matthew J. Gastinger, Bitplane). This XTension employs two alternative computational approaches for defining contact events: a distance transformation method and a surface mask overlap method. For the present analysis, the distance transformation method was selected as the primary detection algorithm. The distance transformation algorithm operates by first computing a three-dimensional Euclidean distance map in the extracellular space surrounding the designated target surface object. For each voxel outside the target surface, the algorithm calculates the shortest distance to the nearest target surface boundary. This distance transform generates a continuous spatial field that represents proximity to the target structure throughout the image volume. For the tracked surface population, the algorithm determines the minimum distance between each surface object and the target surface by sampling the distance transform values at the tracked surface boundaries. This approach efficiently measures the closest surface-to-surface distance without requiring direct geometric intersection calculations.

### Contact threshold definition

A contact event was defined when the minimum surface-to-surface distance fell at or below a threshold value of 0 nm, corresponding to direct physical overlap or touching between the two surface objects. This stringent threshold ensures that only instances of immediate spatial proximity or membrane contact were classified as interaction events, excluding near-neighbor associations that do not involve direct structural apposition.

### Statistical output parameters

The XTension generated quantitative statistics for each tracked surface object, including the instantaneous distance to the target surface at each time point. Additional track-level statistics computed by the algorithm included the following: the total number of contact events per track, the percentage of time points in contact, and temporal parameters such as the duration of individual contact events. These metrics enabled comprehensive characterization of the dynamic spatial relationships between the two surface populations throughout the imaging time series.

### Implementation details

All contact quantification analyses were performed using the distance transformation method within Imaris 10, interfacing with MATLAB through the Imaris XTension framework. The distance transform was computed separately for each time point in multidimensional datasets, and contact statistics were aggregated across the entire time-lapse sequence. The computational workflow ensures reproducible, objective quantification of surface-surface associations without user bias in defining contact criteria.

### Proximity ligation assay

The PLA was performed using Duolink *in situ* Far Red kit reagents (DUO92013, Sigma-Aldrich) according to the manufacturer’s instructions. One lakh fifty thousand LNCaP cells were seeded onto fibronectin-coated glass coverslips. After 24 h, cells were starved in charcoal-stripped RPMI 1640 medium for an additional 24 h, then stimulated with 10 nM DHT for 24 h. Where indicated, cells were treated with TIP for 1 h, followed by a 10-min incubation in ice-cold CSK buffer (10 mM Pipes (pH 6.8) containing 100 mM NaCl, 300 mM sucrose, 3 mM magnesium chloride, 1 mM EGTA, and 0.5% [v/v] Triton X-100).

Fixation was performed at room temperature using 4% PFA in PBS for 10 min. To block nonspecific antibody binding, samples were incubated in 3% BSA in PBS for 30 min at 37 °C in a preheated humidity chamber. Primary antibodies were diluted in a buffer containing 3% BSA and 0.05% Tween-20 in PBS. For codetection experiments, both antibodies were combined in the same buffer. Following removal of the blocking solution, primary antibody mix was applied to each sample and incubated overnight at 4 °C. After gentle PBS washes, PLA probes (anti-mouse MINUS, DUO92004 and anti-rabbit PLUS, DUO92002, Sigma-Aldrich) were diluted in the same antibody buffer and added to the samples. Probe hybridization was carried out for 1 h at 37 °C in a humidity chamber. Posthybridization, slides were washed twice with 1 × wash buffer A (0.01 M Tris, 0.15 M NaCl, 0.05% Tween-20; pH 7.4) for 5 min under gentle agitation. Ligation solution was prepared by diluting ligation stock 1:5 in high-purity water, and ligase was freshly added at a final dilution of 1:40 immediately prior to sample application. Samples were incubated with the ligation mixture for 30 min at 37 °C. Rolling circle amplification was performed by diluting amplification stock 1:5 in high-purity water, with polymerase added at a final dilution of 1:80. Amplification was carried out in the dark for 100 min at 37 °C in a preheated humidity chamber due to the light sensitivity of the reagents. The following primary antibodies were used: mouse monoclonal anti-myosin VI (1:200; Sigma-Aldrich, M0691), rabbit monoclonal anti-DAAM2 (1:200; Abcam, EPR10797(B)), rabbit monoclonal anti-YAP (D8H1X) (1:50; Cell Signaling Technology, 14074S), rabbit polyclonal anti- DAB2 (1:200; Proteintech, 10109-2-AP).

Images were acquired using a Zeiss LSM800 confocal laser-scanning microscope equipped with a 63 × /1.4 NA oil objective, DAPI and Cy5 filter sets, Zen Blue software, and Airyscan Multiplex SR-4Y super-resolution mode. For each sample, Z-stacks comprising 35 optical sections (0.14 μm step size) were collected with DAPI and Cy5 channels. Image analysis was conducted using IMARIS v10.2.0 (Oxford Instruments). Nuclei were rendered in 3D based on the DAPI channel and subsequently masked using the surface masking tool. Cy5-labeled PLA signals were visualized, 3D rendered, and quantitatively assessed within the nuclear compartment across all experimental conditions.

### PSA-luciferase gene reporter assay

LNCaP-wt cells (300.000 cells per well) were seeded in six-well plates in RPMI 1640 medium (Gibco) supplemented with 10% FBS (Biochrome) and 100 units ml^-1^ penicillin/streptomycin (P/S). After 24 h, the medium was removed, and cells were gently washed three times with 1 × PBS. Cells were then starved for 24 h in androgen-depleted medium (RPMI 1640 supplemented with 10% charcoal-stripped FBS and P/S).

Following starvation, cells were transfected using Lipofectamine 3000 (Thermo Fisher Scientific) according to the manufacturer’s protocol. Five hours post-transfection, the medium was replaced with RPMI 1640 supplemented with 10% charcoal-stripped FBS, 10 nM DHT, and 25 μM TIP. Cells were incubated overnight.

For the siRNA-based PSA-luciferase gene reporter assay, 30 pmol siRNA (Qiagen FlexiTube Hs_MYO6_5 for myosin VI knockdown or Qiagen AllStars Negative Control) in Opti-MEM mixed with Lipofectamine RNAiMAX was transfected into cells. The following day, all wells were transfected with 200 ng of pRLTK-Renilla-Luc and 400 ng of pGL3-PSA-Luc per well using Lipofectamine 3000. Five hours later, 2 ml of 20 nM DHT in RPMI 1640 medium supplemented with 10% csFBS and P/S was added dropwise to each well, except for one well treated with 0.1% EtOH. With the remaining 2 ml of medium in the wells, a final concentration of 10 nM DHT was achieved. Twenty-four hours after DHT addition, cells were lysed and luciferase activity was measured.

Medium was aspirated and cells lysed in either 200 μl (six-well plate) or 100 μl (12-well plate) of Triton lysis buffer (0.15 M Tris, 75 mM NaCl, 3 mM MgCl_2_, 0.25% Triton X-100) for 10 min on ice with occasional shaking. Cells were scraped and centrifuged at full speed and 4 °C for 10 min. Next, 20 μl of each lysate was pipetted into a 96-well plate and 50 μl of firefly buffer (15 mM DTT, 0.6 mM coenzyme A, 0.45 mM ATP, 4.2 mg ml^–1^ d-luciferin) was added simultaneously to the lysates and luminescence measured after 10 s. Next, 75 μl of renilla buffer (45 Mm EDTA, 30 mM Na_4_P_2_O_7_, 1.425 M NaCl, 0.06 mM PTC124, 0.01 h-CTZ) was added to stop the reaction and luminescence measured again. Final luciferase values were then determined with the following equation and normalized to the EtOH control:

$$final\;luciferase\;value=\frac{\text{PGL}3\;\text{PSA}-\text{Luc}}{\text{pRLTK}\;\text{Renilla}-\text{Luc}}$$

Afterward, 5 × Laemmli sample buffer was added to the lysates, heated at 95 °C for 10 min, centrifuged for 3 min, and subjected to WB analysis.

#### Cell proliferation and viability assays

Cell proliferation in LNCaP cells was assessed using the Click-iT Plus EdU Alexa Fluor 647 Imaging Kit (Invitrogen). One lakh LNCaP cells were seeded per well in a 24-well glass-bottom plate precoated with 10 μg/ml fibronectin for 60 min to enhance cell adhesion. After 24 h, cells were gently washed with PBS and cultured in charcoal-stripped medium to deplete endogenous hormones. Twenty-four hours later, cells were incubated with 10 μM EdU. Simultaneously, 10 nM DHT and 25 μM TIP were added. After an additional 48 h, cells were fixed, permeabilized, and stained for EdU and DNA according to the manufacturer’s instructions (Thermo Fisher Scientific, 2014). Imaging was performed using a Lionheart FX automated microscope (Agilent BioTek). For each well, 64 fields of view were captured in an 8 × 8 grid and stitched into a composite image. Fluorescence channels were used to detect DAPI (461 nm) and EdU (670 nm). Image analysis was conducted using MetaMorph software, where nuclear areas were identified *via* automated thresholding. The ratio of EdU-positive nuclear area (670 nm) to total nuclear area (DAPI, 461 nm) was calculated to determine the proliferation rate.

Cell viability and proliferation were also assessed using the WST-1 assay kit (Abcam, ab155902; Roche, 2021). WST-1 is a tetrazolium salt that is cleaved by mitochondrial dehydrogenases in viable cells to form a soluble formazan dye. The amount of formazan produced correlates with the number of metabolically active cells and is quantified by measuring absorbance at 450 nm.

For LNCaP cells, 100,000 cells per well were seeded in a 24-well plate. After 24 h, cells were washed with PBS and switched to charcoal-stripped medium. Following another 24 h, cells were treated with DHT and 25 μM TIP. After 48 h of treatment, 50 μl of WST-1 reagent was added to 500 μl of medium (1:10 ratio). Cells were incubated for 1 h, then shaken for 15 s (1 mm amplitude, 173.9 RPM). Absorbance was measured at 450 nm, with 655 nm as a reference wavelength.

For PC3 cells, 40.000 cells per well were seeded in a 24-well plate and treated using the same protocol as for LNCaP cells. After 24 h of DHT and drug treatment, WST-1 reagent was added and absorbance was measured as described above.

Final absorbance values were calculated using the following formula:

Final Absorbance = (Abs_450 xnm_sample − Abs_655 nm_sample) − (Abs_450 nm_blank − Abs_655 nm_blank)

All values were normalized to the negative control (EtOH-treated cells).

### Statistical data analysis

Statistical analyses and graphical representations were carried out using GraphPad/Prismv.10.5.0. Information on statistical tests and their description, and error bars are given in the figure legends. In general, experimental data sets were tested for normality. Statistical significance was evaluated with one-way ANOVA for multiple comparisons (Tukey’s). For a data set with only two variables, an unpaired *t* test was performed. Data are presented as either column bar graph ± sd, scatter dot bar plot with mean ± sd, or violin plots showing all data points with median and quartiles. Statistical significance is indicated as ∗*p* < 0.05, ∗∗*p* < 0.01, ∗∗∗*p* < 0.001, ∗∗∗∗*p* < 0.0001.

## Data availability

All the data are provided in the main manuscript or the supporting information file. Mass spectrometry raw data have been deposited at the ProteomeXchange Consortium (http://proteomecentral.proteomexchange.org) under the accession number PXD068687. Furthermore, all mass spectrometry proteomics datasets used and/or analyzed during this study are available online at the MassIVE repository (http://massive.ucsd.edu/; dataset identifier: MSV000099248). This study did not generate new code for analysis.

## Supporting information

This article contains supporting information.

## Conflict of interest

The authors declare that they have no conflicts of interest with the contents of this article.
