# Supplementary material for: Nuclear myosin VI cooperates with actin to promote transcriptional cluster formation at androgen receptors
Source: J Biol Chem. 2025 Dec 22;302(2):111088. doi: 10.1016/j.jbc.2025.111088 (PMC12858346; doi:10.1016/j.jbc.2025.111088)
Supplement: Supporting Information [file mmc4.docx]

**Supporting information (for Publication)**

**Nuclear Myosin VI cooperates with actin to promote transcriptional cluster formation at androgen receptors**

Irma M. Jayawardana^1,2 *^, Justus M. Fleisch^1^, Julian Knerr^1^, Hong Wang^1,5^, Manuel Holst^1^, Stefan Tholen^3,4^, Oliver Schilling^3,4^, Robert Grosse^1,2,5*^

^1^ Institute of Experimental and Clinical Pharmacology and Toxicology, Medical Faculty, University of Freiburg, Freiburg, Germany

^2^ Spemann Graduate School of Biology and Medicine (SGBM), University of Freiburg, Freiburg, Germany

^3^ Institute for Surgical Pathology, Medical Center – University of Freiburg / Medical Faculty – University of Freiburg, Germany

^4^ Proteomics Platform – Core Facility (ProtCF), Medical Center – University of Freiburg / Medical Faculty – University of Freiburg, Germany

^5^ Centre for Integrative Biological Signalling Studies - CIBSS, University of Freiburg, Freiburg, Germany

*To whom correspondence should be addressed. Email: robert.grosse@pharmakol.uni-freiburg.de

Correspondence may also be addressed to irma.jayawardana@pharmakol.uni-freiburg.de

**Figure S1. (A)** SIM-burst mode timelapse representative image of a NIH3T3 co-transfected with AR-GFP (red) and Halo-Myosin VI (cyan) and nuclear actin chromobody-mCherry (yellow), showing nuclear Myosin VI cluster formation and nuclear actin assembly at the AR after the addition of DHT for 16 h The merged image (far left) highlights co-localization patterns within the nucleus (white dashed outline). Individual channels are shown separately for AR (second panel), Myosin VI (third panel), and actin (fourth panel). Scale bars are indicated. **(B)** Immunofluorescence of proximity ligation assay (PLA) in LNCaP cells. DAPI (nuclei, blue) and Cy5 (PLA dots, white) are shown in cells treated with PLA probes + DHT, Myo6+DHT, DAAM2+DHT, Myo6+ YAP+ DHT and Myo6+ Dab2+DHT. Scale bar: 10 μm. Images are shown as maximum intensity projection (MIP). **(C)** Quantification of nuclear PLA dots. Violin plots show median and interquartile ranges from 15, 10, 12 cells per condition (PLA probes + DHT, Myo6+DHT, DAAM2+DHT, Myo6+ YAP+ DHT and Myo6+Dab2+DHT) per biological replicate (n=3). Triplicate samples shown in three distinct colors, each representing an independent experimental replicate.

**Figure S2 (A)** Violin plot showing the distribution of surface contact percentages between AR and Myosin VI in the presence of DHT (+DHT). Each red dot represents an individual cluster. **(B)** Violin plot showing the number of contact events between AR and Myosin VI clusters under +DHT conditions. **(C)** Time-resolved trajectory overlays of AR and Myosin VI clusters from live-cell SIM imaging. Representative overlays of AR (red) and Myosin VI (cyan) cluster trajectories extracted from live-cell SIM. AR trajectories are rendered in a cyan-to-magenta gradient, while Myosin VI trajectories range from green to yellow, illustrating temporal progression and spatial displacement. These color transitions highlight dynamic colocalization and contact persistence between AR and Myosin VI clusters over time.

**Supplementary Movie 1.** SIM-burst mode timelapse video of a NIH3T3 co-transfected with AR-GFP (red) and Halo-Myosin VI (cyan), showing nuclear Myosin VI at the AR after the addition of DHT for 16 h; Scale bar: 5 μm. Zoomed-in area: 200 μm. Time is shown as ss.ms.

**Supplementary Movie 2.** SIM-burst mode timelapse video of a NIH3T3 co-transfected with AR-GFP (red) and Halo-Myosin VI (cyan) and nuclear actin chromobody-mCherry (yellow), showing nuclear Myosin VI cluster formation and nuclear actin assembly at the AR after the addition of DHT for 16 h; zoomed-in areas and scale bars are indicated. Time is shown as ss.ms.

**Supplementary Movie 3.** SIM-burst mode timelapse video of a NIH3T3 co-transfected with AR-GFP (red) and Halo-Myosin VI (cyan) and nuclear actin chromobody-mCherry (yellow), showing nuclear Myosin VI cluster formation and nuclear actin assembly at the AR after the addition of DHT for 16 h; zoomed-in areas and scale bars are indicated. Time is shown as ss.ms.
