# Supplementary material for: Nuclear myosin VI cooperates with actin to promote transcriptional cluster formation at androgen receptors
Source: J Biol Chem. 2025 Dec 22;302(2):111088. doi: 10.1016/j.jbc.2025.111088 (PMC12858346; doi:10.1016/j.jbc.2025.111088)
Supplement: Figure S1 [file mmc5.pdf]

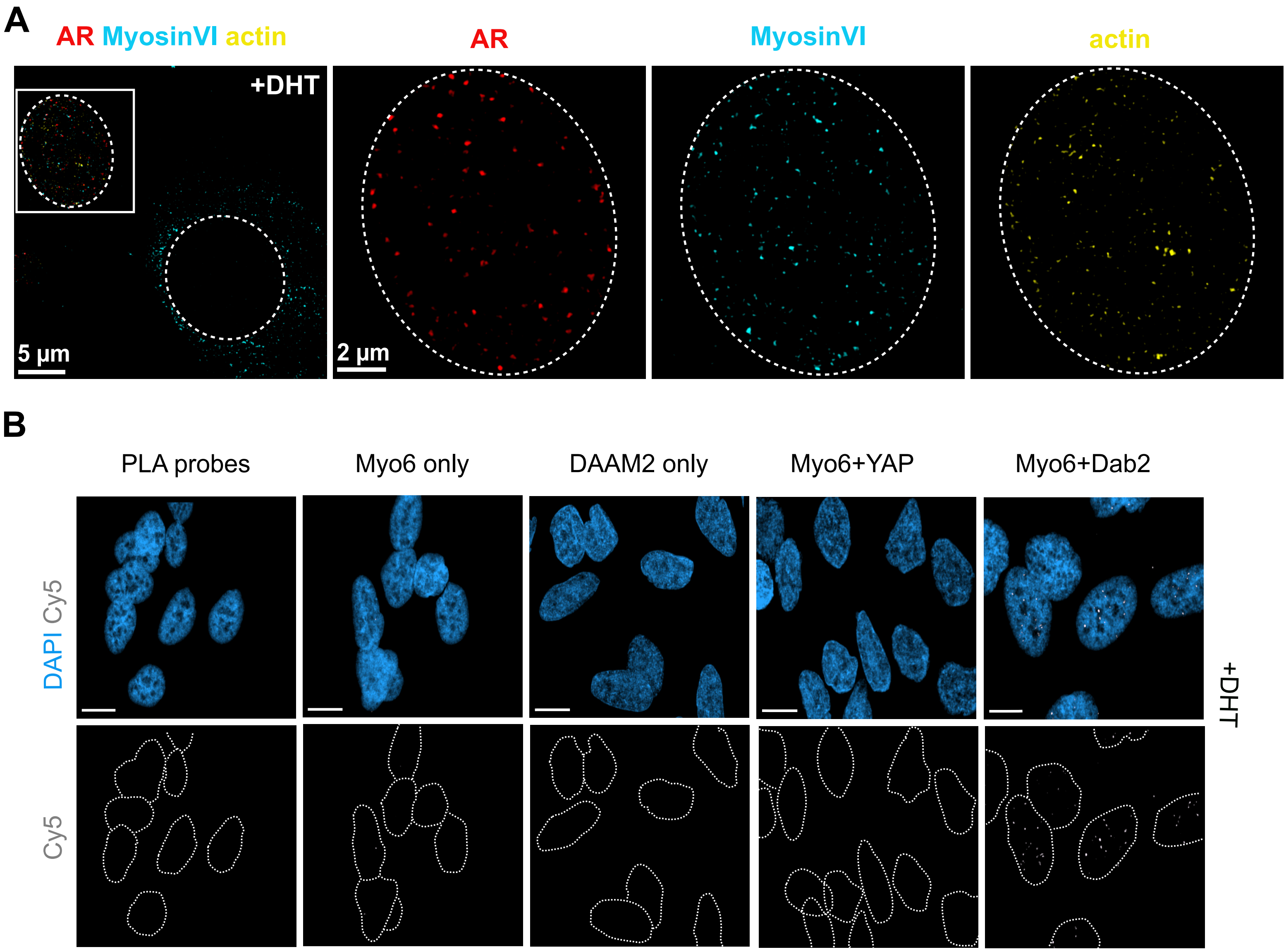

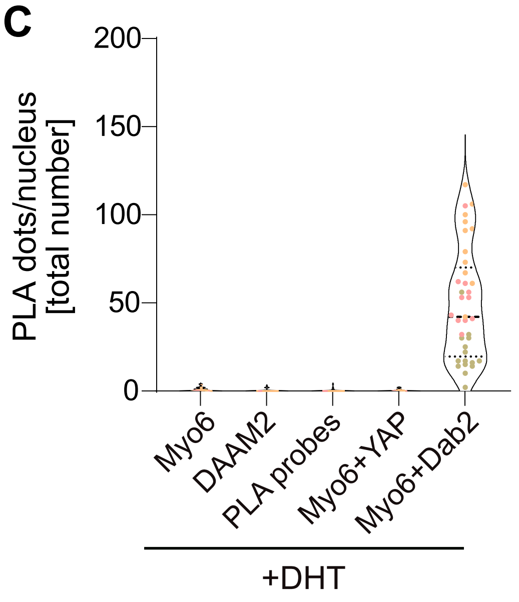

Figure S1. (A) SIM-burst mode timelapse representative image of a NIH3T3 co-transfected with AR-GFP (red) and Halo-Myosin VI (cyan) and nuclear actin chromobody-mCherry (yellow), showing nuclear Myosin VI cluster formation and nuclear actin assembly at the AR after the addition of DHT for 16 h The merged image (far left) highlights co-localization patterns within the nucleus (white dashed outline). Individual channels are shown separately for AR (second panel), Myosin VI (third panel), and actin (fourth panel). Scale bars are indicated. **(B)** Immunofluorescence of proximity ligation assay (PLA) in LNCaP cells. DAPI (nuclei, blue) and Cy5 (PLA dots, white) are shown in cells treated with PLA probes + DHT, Myo6+DHT, DAAM2+DHT, Myo6+ YAP+ DHT and Myo6+ Dab2+DHT. Scale bar: 10 µm. Images are shown as maximum intensity projection (MIP). **(C)** Quantification of nuclear PLA dots. Violin plots show median and interquartile ranges from 15, 10, 12 cells per condition (PLA probes + DHT, Myo6+DHT, DAAM2+DHT, Myo6+ YAP+ DHT and Myo6+Dab2+DHT) per biological replicate (n=3). Triplicate samples shown in three distinct colors, each representing an independent experimental replicate.
