# Supplementary material for: Nuclear myosin VI cooperates with actin to promote transcriptional cluster formation at androgen receptors
Source: J Biol Chem. 2025 Dec 22;302(2):111088. doi: 10.1016/j.jbc.2025.111088 (PMC12858346; doi:10.1016/j.jbc.2025.111088)
Supplement: Figure S2 [file mmc6.pdf]

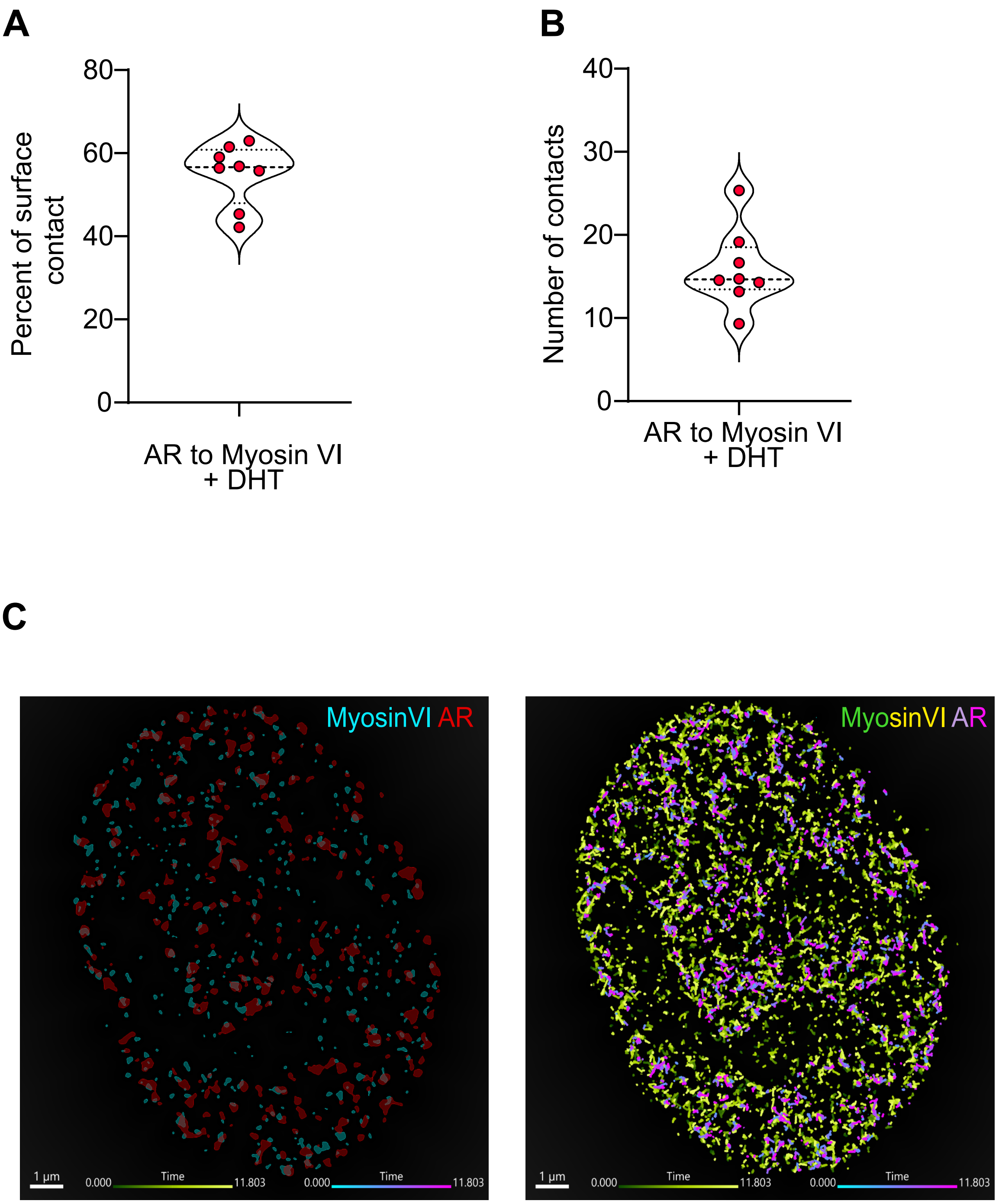

Figure S2 (A) Violin plot showing the distribution of surface contact percentages between AR and Myosin VI in the presence of DHT (+DHT). Each red dot represents an individual cluster. **(B)** Violin plot showing the number of contact events between AR and Myosin VI clusters under +DHT conditions. **(C)** Time-resolved trajectory overlays of AR and Myosin VI clusters from live-cell SIM imaging. Representative overlays of AR (red) and Myosin VI (cyan) cluster trajectories extracted from live-cell SIM. AR trajectories are rendered in a cyan-to-magenta gradient, while Myosin VI trajectories range from green to yellow, illustrating temporal progression and spatial displacement. These color transitions highlight dynamic colocalization and contact persistence between AR and Myosin VI clusters over time.
